# Prediction and validation of total and regional skeletal muscle volume by using anthropometric measurements in prepubertal Japanese children

**DOI:** 10.1017/S0007114522003269

**Published:** 2023-07-28

**Authors:** Taishi Midorikawa, Megumi Ohta, Yuki Hikihara, Suguru Torii, Shizuo Sakamoto

**Affiliations:** 1 College of Health and Welfare, J.F. Oberlin University, 3758 Tokiwamachi, Machida, Tokyo 194-0294, Japan; 2 Waseda Institute for Sport Sciences, Waseda University, 2-579-15 Mikajima, Tokorozawa, Saitama 359-1192, Japan; 3 Faculty of Liberal Arts and Sciences, Chukyo University, 101-2 Yagoto Honmachi, Showa-ku, Nagoya-shi, Aichi 466-8666, Japan; 4 Faculty of Creative Engineering, Chiba Institute of Technology, 2-1-1 Shibazono, Narashino, Chiba 275-0023, Japan; 5 Faculty of Sport Sciences, Waseda University, 2-579-15 Mikajima, Tokorozawa, Saitama 359-1192, Japan; 6 Faculty of Sport Science, Surugadai University, 698 Azu, Hanno, Saitama 357-8555, Japan

**Keywords:** Skeletal muscle volume, MRI, Children, Anthropometry, Prediction equation‘

## Abstract

It is difficult to easily estimate skeletal muscle (SM) volume in children. We aimed to develop regression-based prediction equations to estimate the total body and regional SM volume using calliper measurements of skinfold thickness and limb circumference and to investigate the validity of these equations. In total, 142 healthy, prepubertal, Japanese children, aged 6–12 years, were divided into two groups: the model development group (sixty boys, thirty-eight girls) and the validation group (twenty-six boys, eighteen girls). Contiguous magnetic resonance images were obtained from the first cervical vertebra to the ankle joints as reference data. SM volume was calculated from the summation of the digitised cross-sectional areas. Limb and waist circumferences were measured at mid-upper arm, mid-thigh, maximal calf and at the level of umbilicus. Each girth was corrected for subcutaneous adipose tissue thickness, as estimated by skinfold thickness measurements. Skinfold thickness was measured at the posterior upper arm, anterior thigh, medial calf and lateral to the umbilicus, using callipers. Significant correlations were observed between the site-matched SM volume, measured by MRI, and each corrected girth × standing height value in the model development group. When these SM volume prediction equations were applied to the validation group, the measured total body and regional SM volume were similar to the predicted values. These results suggest that the anthropometric prediction equations developed in this study provide reliable information about the total and regional SM volume in prepubertal Japanese children, with varying degrees of estimation accuracy for each region.

Anthropometric measurements are essential to understand physiological characteristics and estimate body composition. As one of the parameters for estimating skeletal muscle (SM) volume and mass, the corrected girths, which are calculated using the circumference and skinfold thickness, have been used in adult populations^(1,2)^. This parameter can be obtained in a short time using only a tape measure and skinfold callipers, and it requires no other equipment or electricity. Therefore, applying prediction equations using corrected girths would be an effective method for gathering data about SM volume and mass in large-scale studies. The accurately measured SM volume is linked to the meaningful assessment of nutritional status and the physical activity level, thereby helping in the understanding of development and growth in children.

There is currently only one SM mass prediction equation that uses the corrected girths in children. This was used in a study by Poortmans *et al*., which was designed to establish formulas for total body SM mass in children^(3)^. Poortmans *et al*. report that the SM mass can be estimated with satisfactory confidence by simple anthropometric measurements including the corrected girths^(3)^. However, the study had the following limitations: a small sample size (*n* 39; aged 7–16 years), lack of a validation study and the use of appendicular lean soft tissues by dual-energy X-ray absorptiometry (DXA) instead of MRI as reference data.

At the present time, MRI is the most precise, reliable and safe method for the measurement of total and regional SM volume in children^(4–6)^. We have previously developed the prediction equations using ultrasonography and DXA for the estimation of total and regional (i.e. arm, trunk, thigh and lower leg) SM volume and mass in children using MRI as a reference^(7,8)^. The development of the corrected girths-derived SM volume equations, using MRI as a reference, would increase the number of methods for SM volume estimation in children. Thus, the present study was performed to develop and validate regression-based prediction equations for total and regional SM volume using corrected girths in prepubertal Japanese children.

## Experimental methods

### Subjects

In total, 142 healthy, prepubertal Japanese children, aged 6–12 years (determined according to the number of years since their birth) and at Tanner stage 1 were randomly separated into two groups at a ratio of about 2:1 (model development group: validation group) with consideration to the distribution of the overweight and obese in each age group by the international cut-off points for BMI^(9)^. The subjects were divided into the model development group (sixty boys, including nine overweight and three obese boys; thirty-eight girls, including twelve overweight girls) and the validation group (twenty-six boys, including six overweight and one obese boy; eighteen girls, including five overweight girls) (Table 1)^(9)^. The subjects were recruited through referral by friends and acquaintances and through flyers in Tokyo. At the time of enrolment, the criteria for inclusion (i.e. demographic and socio-economic status) were not defined, so that a number of children with varying statuses could be included in this research. The maturation level of the subjects was assessed by authors TM and MO through conversations using the Tanner scale questionnaire^(10)^. All the subjects were physically active (i.e. they played outdoor games); however, the sample did not include any athletes. None of the subjects had any known pathological condition nor were they on any medication. This study was conducted according to the guidelines laid down in the Declaration of Helsinki, and all procedures involving human subjects/patients were approved by the Ethical Committee of Waseda University. Written informed consent was obtained from all subjects and their guardians.

**Table 1.** Subject characteristics and anthropometric parameters (Mean values and standard deviations)

	Development				Validation			
	Boys (*n* 60)		Girls (*n* 38)		Boys (*n* 26)		Girls (*n* 18)	
	Mean	sd	Mean	sd	Mean	sd	Mean	sd
Age (year)	9	2	9	2	9	1	9	2
Standing height (cm)	136·7	11·1	133·1	11·4	136·2	9·3	134·7	11·8
Body weight (kg)	33·1	8·2	31·1	8·7	33·7	9·6	31·8	9·5
BMI (kg/m^2^)	17·4	2·6	17·2	2·7	17·9	3·3	17·3	3·1
Fat (%)	24·1	8·3	28·3	6·7	24·4	8·6	28·0	7·3
Circumferences (cm)								
Mid-upper arm	21·5	3·5	21·4	3·2	21·4	3·9	21·5	3·0
Waist	61·9	8·8	60·4	8·6	63·2	10·9	60·5	9·7
Mid-thigh	39·4	4·9	38·7	5·0	39·5	5·7	39·3	5·0
Calf	28·8	3·0	28·1	3·1	28·9	3·5	28·5	3·1
Skinfold thicknesses (cm)								
Posterior upper arm	1·5	0·7	1·5	0·6	1·4	0·7	1·6	0·7
Abdomen	1·4	1·1	1·4	0·8	1·5	1·2	1·4	0·8
Anterior thigh	1·9	0·8	1·9	0·6	1·9	0·8	2·0	0·6
Medial calf	1·4	0·6	1·4	0·6	1·3	0·7	1·5	0·5
Corrected girths (cm)								
Mid-upper arm	16·9	2·0	16·6	1·8	16·9	2·1	16·5	1·6
Waist	57·6	6·0	56·0	6·7	58·5	7·6	56·0	7·6
Mid-thigh	33·6	3·7	32·8	3·7	33·5	3·7	33·0	4·2
Calf	24·6	2·3	23·7	2·3	24·8	2·1	23·9	2·2

### Anthropometric measurements

Body weight was measured on a digital balance to the nearest 0·1 kg (DC-320, TANITA Co. Ltd), with the subjects wearing only minimal clothing. Standing height was measured on a stadiometer to the nearest 0·1 cm (YS-OA, AS ONE Co. Ltd). BMI was calculated as body weight in kilograms per square metre of the standing height (kg/m^2^) (Table 1). Body fat percentage was measured using whole body DXA scan (Delphi A-QDR, Hologic Inc.; Version 12.4:3 Pediatric Whole body) (Table 1).

Skinfold thickness was measured to the nearest 0·5 mm using Eiken skinfold callipers (MK-60, Yagami). Measurements were recorded at the posterior upper arm, abdomen, anterior thigh and medial lower leg, and on the right side of the body by authors TM and MO who specialise in body composition^(11)^. The four anatomical landmarks for the selected sites were defined as follows: ‘*posterior upper arm*’ was located on the posterior surface of the upper arm (i.e. triceps skinfold thickness), at 50 % distal between the lateral epicondyle of the humerus (near the elbow) and the acromial process of the scapula (at the shoulder); ‘*abdomen*’ was located 2–3 cm lateral to the umbilicus; ‘*anterior thigh*’ was located on the anterior surface of the upper leg, midway between the lateral condyle of the femur (near the knee) and the greater trochanter of the femur (at the hip); and ‘*medial calf*’ was located on the medial aspect of the lower leg, at 30 % proximal between the lateral malleolus of the fibula (near the ankle) and the lateral condyle of the tibia (near the knee).

Circumference measurements were also obtained to the nearest 1 mm at each of the aforementioned anatomical landmarks of skinfold thickness. Measurements were obtained at the plane orthogonal to the long axis of the body segment in question. As indicated by Martin *et al*. (C_m_ = C_limb_ – πS; C_m,_ the corrected muscle circumferences; C_limb,_ the limb circumferences; S, skinfold calliper measurements), the corrected girths (i.e. corrected arm girth, corrected waist girth, corrected thigh girth, corrected calf girth) were calculated from the skinfold thickness and circumference measurement of each landmark^(1)^.

### Skeletal muscle volume measured by MRI

The total body SM volume was measured using a General Electric Signa EXCITE VI 1·5-Tesler scanner. A T1-weighted, spin-echo, axial-plane sequence was performed with a repetition time of 500 ms during breath-holding scans and normal-breathing scans and an echo time of 13·1 ms. The subjects rested quietly in the magnet bore in the supine position, with their hands placed on their abdomen. For each subject, contiguous transverse images with a slice thickness of 1·0 cm (interslice gap: 0 cm) were obtained from the first cervical vertebra to the malleolus lateralis. Approximately five sets of acquisitions were obtained, extending from the first cervical vertebra to the femoral head with breath holding (approximately 20 s per set). The other sets of acquisitions were obtained from the femoral head to the ankle joints during normal breathing^(4)^. All images (approximately 100–150 slices per subject) were traced from the SM segment, excluding the connective tissue, blood vessels, fat tissue and abdominal organs, by a highly trained technician. Magnetic resonance images were analysed by ZedView software (LEXI Co., Ltd.) for segmentation and calculation of cross-sectional tissue areas.

SM volume was calculated by the sum of the cross-sectional area (cm^2^), which was determined by tracing the images and then multiplying the cross-sectional area with the slice thickness (cm). The estimated coefficient of validation for SM volume measurements from a test–retest analysis was determined to be 2 %^(4)^. The SM volume was also separated into discrete regions by using anatomical landmarks that were visible in the scanned images. These are as follows: arm, from the axillary fossa to the styloid process of the radius; trunk, from the first cervical vertebra to the femoral neck; thigh, from the femoral neck to the articular surface of the medial condyle; and lower leg, from the articular surface of the medial condyle to the malleolus lateralis.

### Statistics

All the results are presented as means and standard deviations. To make the SM volume prediction equations, simple regression analyses were used to describe the relationship between the MRI-measured SM volume and each squared corrected girth × height, at each regional area, for the model development group (i.e. the sample size was justified for both boys (*n* 60) and girls (*n* 38) with a correlation parameter of 0·9 using power analysis). Moreover, stepwise regression analysis was performed with each squared corrected girth (i.e. corrected arm girth, corrected waist girth, corrected thigh girth and corrected calf girth) × height as independent values at total body SM volume. As indicated in a previous study^(2)^, since total and regional SM are conceptually in the form of a cylinder, the squared corrected girth and standing height were used to express a predictor of SM area and length to match the cylinder’s dimensions.

For the validation group, the difference between the measured and predicted SM volume was examined using paired *t* tests, Cohen’s d and Lin’s concordance correlation coefficient (i.e. the sample size was justified for both in boys (*n* 26) and girls (*n* 18) using power analysis). Furthermore, the agreement between the measured and predicted values of SM volume was examined by plotting the differences against the means with the limits of agreement as suggested by Bland and Altman (mean difference (2 sd) of the difference: 95 % limits of agreement). This gives an indication of the precision of the method^(12)^. Statistical analyses were performed using SPSS for Windows (IBM SPSS version 27.0; SPSS Inc.) and MedCalc (version 16.2.0; MedCalc Software bvba, Belgium). Differences were considered significant when the *P* value was < 0·05.

## Results

The physical characteristics and anthropometric parameters are summarised in Table 1. The mean standing height and body weight values were comparable to those found in ‘Physical fitness standards of Japanese people’ by Tokyo Metropolitan University Press^(13)^, indicating that the volume and distribution of SM in the subjects of the present study are representative of that found in prepubertal Japanese children.

Regression analysis found significant correlations between the site-matched SM volume (total body, arm, trunk, thigh and lower leg) measured by MRI and the squared corrected girth × standing height in the model development group, for both boys and girls (Table 2).

**Table 2.** The predictive equations for MRI-measured total body and regional skeletal muscle volume using CAG, CWG, CTG and CCG

Skeletal muscle volume (cm^3^)	Equation	*R*_2_^adj^	SEE
Boys (*n* 60)			
Total	SMV_MRI_ = 2·563 × CTG^2^ × Ht + 5·163 × CCG^2^ × Ht + 598·210	0·89	758
Arm	SMV_MRI_ = 1·495 × CAG^2^ × Ht + 228·162	0·73	114
Trunk	SMV_MRI_ = 0·569 × CWG^2^ × Ht + 874·080	0·61	553
Thigh	SMV_MRI_ = 1·963 × CTG^2^ × Ht + 399·516	0·83	396
Lower leg	SMV_MRI_ = 1·278 × CCG^2^ × Ht + 86·570	0·86	111
Girls (*n* 38)			
Total	SMV_MRI_ = 3·101 × CTG^2^ × Ht + 4·162 × CCG^2^ × Ht – 83·404	0·91	651
Arm	SMV_MRI_ = 1·638 × CAG^2^ × Ht + 97·462	0·82	83
Trunk	SMV_MRI_ = 0·551 × CAG^2^ × Ht + 584·044	0·69	474
Thigh	SMV_MRI_ = 1·943 × CTG^2^ × Ht + 105·872	0·86	340
Lower leg	SMV_MRI_ = 1·353 × CCG^2^ × Ht + 6·033	0·89	96

Total, total skeletal muscle volume of arm, trunk, thigh and lower leg regions; *R* ^2^_adj_: adjusted *R*-squared; SEE, standard error of the estimate; CAG, corrected arm girth; CWG, corrected waist girth; CTG, corrected thigh girth; CCG, corrected calf girth; Ht, standing height (m).

**Fig. 1.**
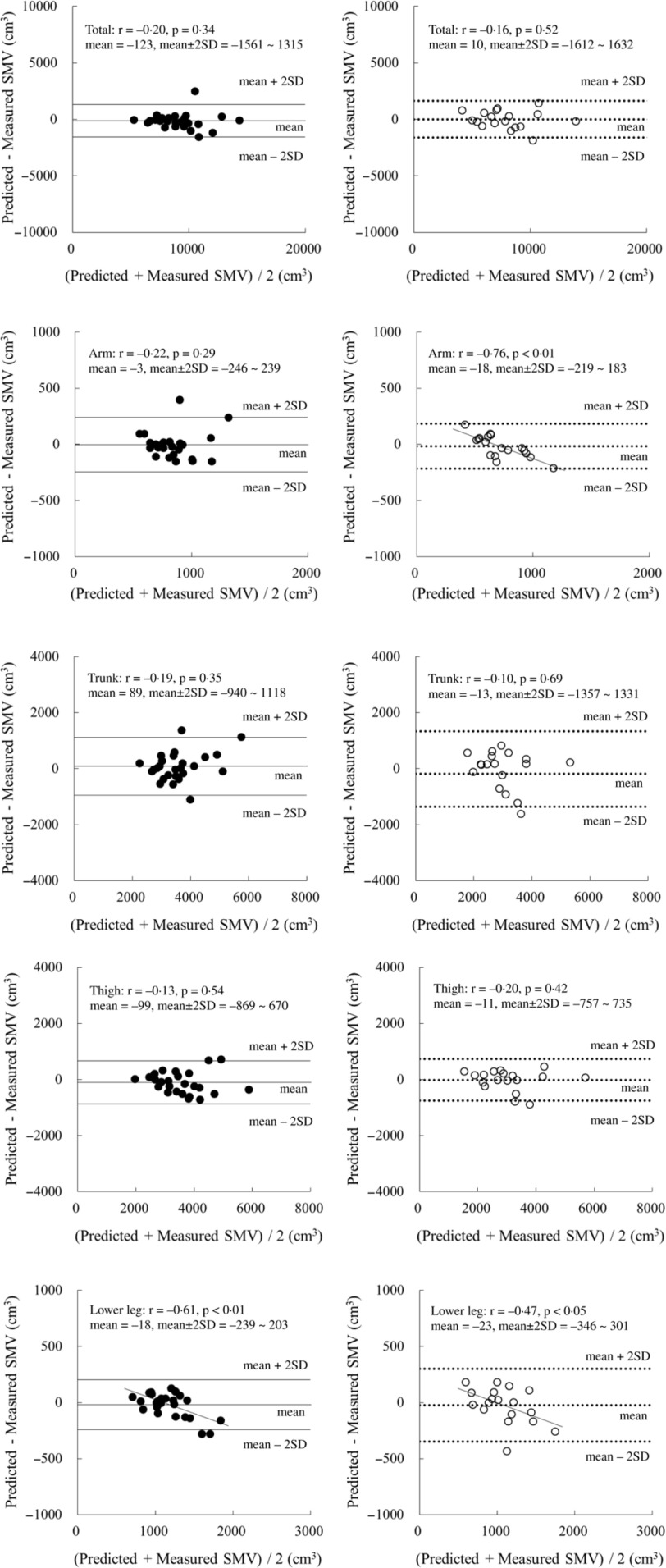
Bland–Altman analysis for the validation group. ●: boys (n 26), ○: girls (*n* 18). Mean ± 2 sd: Solid line, boys; dotted line, girls.

When these SM volume prediction equations were applied to the validation group, the measured total and regional SM volumes were similar to their respective predicted values for both boys and girls (Table 3). Moreover, concordance correlation coefficient of the total and thigh values was higher than that of the arm and trunk values (Table 3). The results of the Bland–Altman analysis for the validation group did not indicate any bias for either boys or girls, with the exception of the arm region for girls and the lower leg region for both boys and girls (Fig. 1).

**Table 3.** The measured and predicted skeletal muscle (SM) volume in total body and regional segments for validation in boys and girls (Mean values and standard deviations)

Skeletal muscle volume (cm^3^)	Boys (*n* 26)									Girls (*n* 18)								
	Measured		Predicted		Mean difference		*p*	*d*	CCC	Measured		Predicted		Mean difference		*p*	*d*	CCC
	Mean	sd	Mean	sd	Mean	sd				Mean	sd	Mean	sd	Mean	sd			
Total	9101	2123	8978	2071	–123	719	0·41	–0·06	0·94	7858	2521	7868	2391	10	811	0·96	0·00	0·95
Arm	832	193	829	198	–3	121	0·99	0·00	0·81	725	237	707	162	–18	100	0·46	–0·09	0·87
Trunk	3516	763	3604	907	89	514	0·39	0·11	0·81	2989	955	2976	829	–13	672	0·94	–0·01	0·72
Thigh	3566	917	3466	870	–99	385	0·20	–0·11	0·90	3055	1038	3045	991	–11	373	0·90	–0·01	0·93
Lower leg	1187	311	1170	244	–18	111	0·56	–0·04	0·93	1089	350	1066	275	–23	162	0·56	–0·07	0·87

Total, total skeletal muscle volume of arm, trunk, thigh and lower leg regions; d, Cohen’s *d*; CCC, Lin’s concordance correlation coefficient between measured and predicted SM volume.

Mean difference: calculated as (predicted – measured SM volume).

*P* value for paired *t* tests: measured v. predicted SM volume.

## Discussion

Although SM volume is a very effective index for nutrition and sport sciences, the precise and safe estimation of SM volume in children is still difficult. In fact, information about total body SM volume/mass in children using MRI was limited. Hsu *et al*. assessed the relationship between resting energy expenditure and organ-tissue body composition such as SM mass and internal organs mass in fifteen children (eight boys and seven girls aged 6–12 years)^(14)^. Kim *et al*. developed prediction equation of total body SM mass using DXA in eighty-three children (forty-six boys and thirty-seven girls aged 5–14 years)^(15)^. However, till date, there is no large-scale MRI study on assessment of growth of SM mass in children except our previous research^(7,8)^.

Given this, we developed prediction equations using corrected girths while overcoming the limitations of the previous study by Poortmans *et al*.^(3)^ To overcome these limitations, we included a relatively large number of subjects and a validation group, and we used MRI as reference data^(3)^. Our present prediction equations for total body SM volume displayed a relatively high *R* ^2^_adj_ value, a moderate standard error of the estimate (SEE) and an excellent concordance correlation coefficient for the validation group. Our previous prediction equations using DXA, which had a quick scan and analysis but a low radiation exposure, had a higher *R* ^2^_adj_ value (boys 0·97; girls 0·97) and a lower SEE (boys 423 cm^3^; girls 413 cm^3^)^(8)^. Nevertheless, the *R* ^2^_adj_ value and SEE in the present study are similar to those obtained using our previous equations (*R* ^2^_adj_ value: boys 0·93; girls 0·89 and SEE: boys 659 cm^3^; girls 731 cm^3^) using ultrasonography, which was non-invasive but needed a device^(7)^. Therefore, the current prediction equations are generally useful for estimating the total body SM volume in prepubertal children for whom ultrasonography results are not available. In addition, Ohta *et al*. indicated that the ratio of body weight to waist circumference at the level of umbilicus can be a convenient measure for assessing the total body SM volume in prepubertal children (*R* ^2^ value: boys 0·94; girls 0·94 and SEE: boys 706 cm^3^; girls 825 cm^3^)^(16)^. Anthropometric measurements using a tape measure would expand the options for obtaining precise total body SM volume data of prepubertal children in field research.

In the present study, it was highlighted that the prediction equations were developed for each anatomical region in children. The results obtained could lead to an understanding of the distribution of SM mass in the growth stage. However, the accuracy of the predicted SM volume estimations of the arms was low with our present prediction equations. Possibly, the small mass of SM (i.e. around 1 kg) led to a large variation in the estimations. Moreover, it was speculated that the underestimation of a large arm’s SM mass in girls might be caused by an overestimation of the triceps skinfold thickness. In fact, the low accuracy of the SM prediction equation for arm was also observed in our previous study using ultrasonography (*R* ^2^_adj_ value: boys 0·71; girls 0·80 and SEE: boys 124 cm^3^; girls 89 cm^3^)^(7)^. In addition, previous DXA-derived prediction equations showed limited success in terms of accuracy (*R* ^2^_adj_ value: boys 0·90; girls 0·86 and SEE: boys 77 cm^3^; girls 77 cm^3^; bias of the Bland–Altman analysis in girls for the validation group)^(8)^. Thus, the predicted SM values for arms should be used only as a guide, even when there is access to DXA.

The corrected girth value for the trunk has not been adopted in previous studies in children and adults^(1–3)^, since the trunk consists of not only SM, fat and bone but also internal organs. The present study attempted to derive a prediction equation for trunk SM volume using corrected waist girth values in prepubertal children. The *R* ^2^_adj_ value was lower and the SEE value was higher in the current study than in our previous DXA-derived prediction equation study (*R* ^2^_adj_ value: boys 0·96; girls 0·86 and SEE: boys 192 cm^3^; girls 317 cm^3^)^(8)^. However, the prediction model in the present study yielded a similar *R* ^2^_adj_ value and SEE compared with those obtained in our previous ultrasonography-derived prediction equations (*R* ^2^_adj_ value: boys 0·65; girls 0·57 and SEE: boys 565 cm^3^; girls 561 cm^3^)^(7)^. Based on these accuracy estimations, DXA-derived prediction equations are superior for assessing the trunk SM volume in prepubertal children.

Legs are mainly composed of SM, fat and bone. They have a SM mass of around 4–5 kg in prepubertal children^(7,8)^. The present prediction equations for thigh and lower leg SM volumes have higher estimated accuracy than those for the arm and trunk do. This trend is also seen in our previous ultrasonography-derived and DXA-derived prediction equations^(7,8)^. When estimating the SM volume of the leg region, the choice of prediction equations used for prepubertal children depends on the availability of equipment. Additionally, when lower leg measurements in both boys and girls were applied in the current equations, the resulting values were found to be underestimations for children with a larger SM volume. Since a significant correlation was noted between the difference of predicted minus measured SM volume and body fat percentage in boys (*r* = –0·57, *P* < 0·01), the overestimation of skinfold thickness might be one of explanations for this bias of the Bland–Altman analysis. Although it was not applied in girls, this bias needs to be considered during the application of these equations.

The current study had four limitations. First, since previous studies about prepubertal children’s regional SM volume using MRI were very limited, in the present study we could only use our previous reports for comparison. Second, because our corrected girth-derived prediction equations were developed for healthy, prepubertal Japanese children, these equations may not apply to adolescents, children with a disease or children from other countries. Third, the prediction equations were suitable for application at the group level, but further work is needed to improve the accuracy of the prediction equations when applied at the individual level. Fourth, since the measurements of circumference and skinfold thickness were performed by two body composition specialists to shorten measurement time and with attention to sex, the possibility of inter-observer variability cannot be ruled out.

In the present study, we developed precise and accurate corrected girth-derived prediction equations for the estimation of total and regional SM volume in prepubertal boys and girls. When combined with our previous total body fat prediction model^(17)^, one could estimate the SM volume and fat mass concurrently. Therefore, the current prediction equations could increase the available options for the estimation of body composition in children. The ease of applicability in field settings is also an advantage.
